# Curiosity in calamity: How personal schadenfreude shapes disaster-tourism intentions

**DOI:** 10.1371/journal.pone.0347535

**Published:** 2026-05-28

**Authors:** Joshin Joseph, Mahmoud M Abdelwahab, Ibrahim Elbatal, Jiju Gillariose, Mustafa M. Hasaballah

**Affiliations:** 1 School of Commerce and Professional Studies, Marian College Kuttikkanam, Peermade, Kerala, India; 2 Department of Mathematics and Statistics, Faculty of Science, Imam Mohammad Ibn Saud Islamic University (IMSIU), Riyadh, Saudi Arabia; 3 Department of Mathematics and Statistics, Faculty of Science, Imam Mohammad Ibn Saud Islamic University (IMSIU), Riyadh, Saudi Arabia; 4 Department of Statistics and Data Science, Christ University, Bangalore, Karnataka, India; 5 Department of Basic Sciences, Marg Higher Institute of Engineering and Modern Technology, Cairo, Egypt; Guilin University of Technology, CHINA

## Abstract

Tourism to sites of recent disaster, a form of dark tourism has raised questions about whether visitors are driven by typical travel motivations or by morbid impulses. This study examines how conventional tourist motives and the personality trait of benign schadenfreude (pleasure at others’ misfortune) jointly influence people’s intentions to visit a recent disaster site. By surveying 438 tourists to Kerala, four months after the July 2024 Wayanad landslides, we measured four common travel motives (novelty seeking, fun/entertainment, knowledge/learning, and relationship bonding) alongside a benign schadenfreude scale and visit intention. Partial least squares structural equation modelling (PLS-SEM) was employed for modeling. The model explained 58.80 percent of the variance in visit intention. Three motives viz., novelty, knowledge, and relationship had significant positive associations with intention, whereas the fun motive showed a negative effect. Schadenfreude emerged as the strongest predictor of disaster-site visit intention. Moreover, schadenfreude significantly moderated the influence of novelty seeking: respondents high in schadenfreude exhibited especially strong curiosity-driven intent to visit. These findings suggest that interest in post-disaster tourism often stems from ordinary travel drivers (curiosity, learning, social bonding), but a disposition to enjoy others’ misfortune can intensify the appeal when novel experiences are involved. The research highlights the need for ethical considerations to be followed by the destination managers and authorities in managing dark tourism destinations. Key limitations include the use of a cross-sectional data, region-specific sample and the focus on benign dimension (versus malicious) of schadenfreude. Future research should validate these results in other cultural and disaster contexts, establish causal relationships, and examine additional personal factors as well as dimensions of schadenfreude.

## Introduction

The landslide that struck on Wayanad, Kerala, India on 30^th^ July was a catastrophic series of earthflows triggered by heavy monsoon rains transformed a tranquil tourist paradise into a scene of devastation [1]. In total, the disaster has claimed 392 human lives, with around 150 people missing and hundreds injured [2]. Wayanad, a geographical region recognised for its lush green hills and popular resorts, saw entire villages like Mundakkai and Chooralmala reduced to rubble [1]. This tragedy created a site of immense human suffering – yet also one of intense public interest. In the aftermath, questions arose about “disaster tourism”: What would motivate someone to visit such a site of recent tragedy? Can the same mainstream tourist motives that draw travellers to scenic or cultural destinations (e.g., curiosity, leisure, learning, social bonding) also explain an intention to visit a disaster location? Or does such interest veer into the territory of “dark tourism,” driven by morbid fascination or even schadenfreude (pleasure at others’ misfortune)?

Tourism to sites of death, disaster, and tragedy often termed dark tourism or thanatourism has become a notable phenomenon in recent decades [3]. From the memorials of Ground Zero and Auschwitz to the ruins of natural disaster zones like Chernobyl or tsunami-hit regions, millions of travellers are drawn to places associated with catastrophic loss of life [3]. Scholars and observers have long debated what motivates people to visit such morbid destinations. Dark tourism can teach about past tragedies and let people pay respects, but some visitors go out of morbid curiosity or even pleasure in others’ pain [4,5]. Understanding these motives helps explain tourist behaviour, run sites ethically, and address public concerns.

People visit disaster sites to learn, honour history, and reflect [6]. For instance, visiting a genocide museum or a natural disaster memorial can be driven by a desire to learn about what happened and why, or by commemorative motives the intention to remember and honour the dead [6]. At the same time, researchers acknowledge that darker impulses might also play a role. Classic literature on thanatourism has pointed out “morbid curiosity” and the lure of the macabre as powerful pull factors for some tourists [5]. Indeed, certain “unconventional” motives identified in dark tourism research include voyeurism (a fascination with seeing the gruesome), curiosity about death, and even schadenfreude, meaning a covert pleasure in witnessing sites of others’ suffering [5]. While such motives are seldom openly admitted by tourists (due to social desirability and moral norms), they have been theorized as underlying drivers that differentiate dark tourism from other heritage or cultural tourism experiences [7]. The present study seeks to explore these complex motives – both “light” (e.g., education, remembrance) and “dark” (e.g., curiosity, thrill-seeking, schadenfreude) – and how they relate to a travellers’ intention to visit disaster locations.

Crucially, we focus on the role of personal schadenfreude as a moderating factor in dark tourism motivation. Schadenfreude refers to the sense of satisfaction or joy at another’s misfortune [8]. Although it is a socially disapproved emotion, it is also a common human experience that psychologists have studied in various contexts (from workplace rivalries to sports fandom). In the tourism domain, schadenfreude might manifest, for example, as a grim thrill or satisfaction a person feels when viewing a place where others have fallen victim to tragedy. Prior conceptual work has indeed suggested that a subset of dark tourists sometimes dubbed “‘schadenfreude’ tourists” derive a secret pleasure from seeing scenes of calamity [5]. If this is true, individuals who have a higher dispositional tendency to experience schadenfreude may be more drawn to disaster sites, or may experience the usual motives (like curiosity or thrill) in a different way. However, empirical evidence on this point remains limited. This research aims to fill that gap by examining whether schadenfreude-prone individuals exhibit stronger intentions to visit disaster sites, and whether schadenfreude alters the influence of other motives on those visit intentions. From theoretical perspective, exploring schadenfreude in dark tourism can answer broader questions of ethical consumption of a tragedy. Dark tourism has often been criticized when tourists appear to treat solemn sites as venues for entertainment or ghoulish excitement alone. Understanding the psychology and logic underlaying theory schadenfreude, which is linked to envy, rivalry, and perceptions of justice; in this context could help to distinguish between tourists who approach sites with respectful remembrance of the event versus those who might be seeking a more perverse gratification. From a practical perspective the site managers and destination marketers can benefit from knowing the motivational profiles of their visitors. If, for example, thrill-seeking and schadenfreude are significant factors, it raises questions about how to design interpretative experiences that channel visitors’ attention in respectful ways and discourage voyeurism or trivialization of suffering [4].

This research addresses these questions by examining the role of standard tourist motivations in driving intentions to visit post-disaster locations, specifically the Wayanad landslide sites, and how these motivations might be moderated by personal schadenfreude. The study is positioned deliberately outside the traditional dark tourism framework: unlike classic dark tourism research that often centres on death related attractions (cemeteries, genocide memorials, etc.) and associated morbid curiosity, our focus is on ordinary travel motives and their applicability in an extraordinary context of a recent natural disaster. By integrating the personality trait of schadenfreude, we probe whether a benign tendency to derive enjoyment from others’ misfortunes can amplify one’s curiosity or willingness to tour a disaster-stricken area.

## 2. Literature review and hypothesis

The proposed theoretical framework of the study is grounded in the Push-pull motivation theory [9] coupled with the trait activation theory [10]. As per push-pull theory of tourist motivation, the tourists are motivated by the psychological impulse which are internal in nature, which is often regarded as push factors and inculcates factors such as the quest for novelty, thrust for knowledge and the belonging for social bonding. While these motives explain the general drive to travel. The trait activation theory advocates that specific personality characteristics such as schadenfreude can act as a latent disposition that are triggered by pertinent situational prompts (in this case, a disaster site). That is, the personality traits influence behavior more strongly when situational cues activate them. Post-disaster contexts constitute salient cues involving human misfortune, uncertainty, and moral ambiguity, which in turn activate schadenfreude-related tendencies. By integrating these theories, the model proposes that while standard ‘push’ motives drive the intention to visit, where the personality trait of schadenfreude moderates this relationship by reducing moral inhibition and amplifying curiosity.

### 2.1. Tourist Motives

Novelty, Enjoyment, Learning, and Relationships; Decades of research in travel behaviours have identified a range of core tourist motivations that consistently push people to seek out new destinations. Foremost among these is the desire for novelty and curiosity. Tourists often travel in search for novelty, to get out of their everyday life [11]. Novelty-seeking is a fundamental driver can spark positive emotions in humans by providing adventure as well as surprise [12]. This is because, Cohen [13] theorized and later researchers confirmed that unfamiliar environments can satisfy an innate curiosity and exploratory urge in the travellers [11]. Correia, Kozak, & Ferradeira [14] identified novelty and curiosity as key motivators for destination choice in leisure travel. These findings suggest that even a disaster site, despite its tragic aspect, can attract visitors out of a curiosity to see something that is dramatically novel or rare (such as the immense landslide scar and its aftermath). Indeed, psychological studies note that people have an “intrinsic interest in the unusual,” which in tourism is a postulate to visit distinctive sites, including extreme geophysical events. Novelty seeking is a primary ‘push’ factor in tourism and is operationalised as the desire to experience the unknown and escape the mundane.

Amusement is the another underlaying motive for tourists [15]. That is travelling in pursuit of entertainment, fun, and enjoyment. The pleasure travel is fundamentally linked with hedonistic rewards. Crompton [15] identified boredom alleviation and disinhibited play as the fundamental factor for travel. Tourists often seek to get engaged with activities and events that are exciting as a technique for stress realise. Though, it is counterintuitive to identify a disaster location as a “fun” site, there might be some visitors who subconsciously frame the visit to the disaster location as a source for adventure, thrill and excitement. The concept of “edutainment” in tourism also shows that entertainment and learning need not be mutually exclusive [16]. Thus, a landslide site could be viewed through a lens of experiential thrill – not unlike chasing storms or volcano eruptions for the exhilaration. Yoo, Yoon, & Park [17] found that intrinsic tourist motivations (analogous to seeking enjoyment or personal enrichment) have a significant positive influence on travel intentions. This implies that individuals who strongly value fun and adventure in travel may be inclined to include unconventional sites in their itinerary if those sites promise a unique, exciting experience. This motive is operationally defined as the pursuit of hedonistic rewards, including excitement, thrill, and disinhibited play.

Tourism also has a well-established educational and learning dimension. Many travellers are motivated by a desire to acquire knowledge, enrich their understanding of history, culture, or the natural world. Novelty & knowledge-seeking together form a major “push” factor for travel [18]. Visiting a place associated with a disaster can satisfy intellectual curiosity – for instance, learning about the geology of landslides, witnessing the power of nature first-hand, or understanding the impact on local communities. Research on heritage and disaster sites shows that tourists often cite education as a motive: they want to learn about what happened and “be part of a larger historical narrative” [19]. In fact, studies of dark heritage tourism (such as visits to battlefields or memorials) reveal that many visitors are primarily seekers of knowledge and understanding, rather than thrill-seekers [19]. Further considering the context of Wayanad, a tourist might justify the visit as an educational pilgrimage, that is because of the intention to see site as a matter for the comprehend the disaster’s magnitude, and perhaps empathize with the event’s significance, which is consistent with Biran et al. [19] that motivations for visiting sites of death and suffering often mirror those in heritage tourism, including a pursuit of authentic learning experiences. Even the visit to a natural disaster site, the mainstream tourists may approach it with the same learning-oriented objective which is applicable to a museum or a historical monument site visit. In this study, the education motive is defined as the tourist’s desire to acquire knowledge, understand the magnitude of the event, and empathize with the historical narrative of the site.

Finally, relationship motives frequently underlie travel decisions. Tourism is often a social activity used to strengthen bonds with family and friends or to meet new people. Crompton’s review identified “reinforcement of bonds with intimate groups”, Crompton [15] essentially spending quality time with loved ones, as a core motive for pleasure travel. Likewise, Yoo et al. [17] discuss a relationship motive, noting that travel experiences can enhance family togetherness or camaraderie among peers. Vacationing in a group provides shared memories that bolster relationships. In the Wayanad case, one might imagine friends or couples deciding to venture to the landslide site together as an unusual but meaningful experience to share. A trip to a disaster-affected area could be framed as an act of mutual exploration (“we faced this eye-opening experience together”), thereby fulfilling a social bonding goal. Even beyond one’s immediate circle, travel can satisfy a need for social interaction; meeting locals or other visitors, discussing the event, etc. Empirical research supports that social/personal connection motives are significant predictors of tourist behaviour [15]. In short, the relationship-oriented motive, whether it is spending time with family or engaging with broader human stories, can be applicable even in post-disaster tourism contexts. The relationship motive is operationally defined as the desire to strengthen social bonds with intimate groups (family/friends) through shared meaningful experiences.

While considering the tourist motives, our first proposition is that mainstream tourist motives viz., novelty/curiosity, entertainment/fun, education/learning, and relationship bonding – will positively influence intention to visit a disaster location. When people consider visiting Wayanad after the landslide, they may do so for the same reasons they visit any destination: to experience something new, to have an interesting/enjoyable trip, to learn from the place, or to share an experience with others. Prior studies (See, Yoo, Yoon, & Park [17]) indicate that such motivations are positively linked to tourists’ visit intentions in general. We therefore expect these motives to also drive disaster site visitation intentions

*H1: Tourist Motives (novelty/curiosity, entertainment/fun, education/learning, and relationship bonding) have a positive relationship with intention to visit a disaster location.*

### 2.2. Disaster Tourism vs. Dark Tourism, and the Intrigue of Misfortune

Dark tourism (thanatourism) involves travel to sites associated with death, suffering, or the ostensibly macabre (e.g., concentration camps, disaster memorials, cemeteries) [20]. It has often been portrayed as motivated by morbid curiosity or even voyeurism – a fascination with death and disaster for its own sake. Indeed, Stone & Sharpley [21] argue that a “fascination with death” is a primary motive drawing people to dark sites. However, recent research complicates this view: many visitors to dark sites report ordinary motives like learning, remembrance, or connecting with heritage rather than ghoulish excitement [19,21]. Biran et al. [19] found that at sites like Auschwitz, dominant motivations included education, curiosity, and paying respects, paralleling those of general heritage tourism (and not necessarily a morbid obsession) [22]. In other words, the demand-side experience of dark tourism might not be so “dark” after all; it can resemble mainstream tourism with an added emotional gravity. Disaster tourism is more specific, visits are motivated by the aftermath of a catastrophic event (natural or human‑made) and the processes of relief, recovery, and reconstruction on the ground [23,24]. Places struck by a recent disaster where visitors observe or participate in the aftermath (e.g., devastated neighbourhoods, exclusion zones, reconstruction tours), with prime intention to witnessing impacts, solidarity, sometimes volunteerism or “reality‑checking”; can include voyeuristic curiosity that raises ethical issues [23,24].

Post-disaster tourism means visiting a place soon after a disaster such as the Wayanad landslide area, people usually go to learn, pay respects, and reflect rather than to enjoy others’ suffering 18. These visits can raise awareness and support local recovery through spending [20]. Kerala’s “Revisit Wayanad” campaign invited tourists back and emphasized that most of the region was safe and beautiful [25]. Overall, this is managed as part of recovery, not thrill-seeking. However, scholars acknowledge that some tourists visit disaster sites not for education or altruism but out of morbid curiosity or the thrill of witnessing misfortune. Seaton & Lennon [26] defined schadenfreude as the pleasure derived from others’ misfortune. Similarly, the Buda & McIntosh [27] discuss “a secret pleasure in witnessing the misfortunes of others” as a driver for certain visitors to sites of death and disaster. Further, Suosheng Wang [5] describes a type of dark tourist labelled the “schadenfreude tourist,” who “glean[s] a secret pleasure from seeing others’ misfortune” [20]. Some visitors treat disaster sites like places for amusement and engage in activities such as taking smiling selfies-which draws public criticism [20].This small group shows that enjoyment of others’ misfortune can influence why some people choose to visit. Rather than label a fixed “type” of tourist, we view schadenfreude as a personal trait that differs across individuals [5]. In our model, this trait acts as a moderator—meaning it changes how strongly motives (e.g., learning, remembrance, curiosity) turn into an intention to visit. When schadenfreude is higher, the same motives are more likely to lead to plans to visit; when it is lower, those motives are less likely to become intentions. This is a simple, rational way to explain mixed behaviours without pathologizing most visitors and helps guide ethical site management. By examining personal schadenfreude as a moderator, we integrate a psychological disposition into the tourism motivation-intention model.

### 2.3. Personal Schadenfreude: Concept and Role in Tourist Intentions

Schadenfreude (a German term meaning “harm-joy”) is defined in psychology as the feeling of pleasure or satisfaction at another’s misfortune [28]. It is considered a complex social emotion, often arising in contexts of rivalry, envy, or when one perceives someone “deserved” their bad luck (feeling justice has been served) [29]. Importantly, schadenfreude personality trait dimension people exhibit individual differences in their propensity to experience schadenfreude. Crysel and Webster [28] introduced a Trait Schadenfreude Scale to measure this disposition, confirming that schadenfreude can be assessed as a stable trait with reliable individual variance [28]. Their scale comprises 12 items loading on two distinct factors: benign schadenfreude and malicious schadenfreude [29]. Benign schadenfreude refers to enjoyment of others’ misfortunes in a more innocent or indirect way, without personal malice. For instance, laughing at a goofy mishap or finding a trivial misfortune amusing. Malicious schadenfreude, by contrast, involves a harsher delight in the downfall of others, often that one envy or dislikes (e.g., rejoicing when a rival fails) [28]. Crysel and Webster describe the difference vividly: “benign schadenfreude is akin to laughing at a video of a cat falling off a counter, whereas malicious schadenfreude is closer to the joy some people experience when others lose their jobs” [28]. In our study, we focus on the benign schadenfreude dimension – the kind of low-level, impersonal pleasure at seeing mishaps that many people might admit to.

### 2.4. Why examine schadenfreude in the context of disaster tourism?

The premise is that a person who has a higher tendency to experience benign delight in others’ misfortunes might be more drawn to visiting a site where misfortune has occurred. Such an individual may have a stronger morbid curiosity and be less inhibited by empathy or moral discomfort when considering touring a disaster scene. Psychological research supports this argument. Trait schadenfreude has been found to correlate negatively with empathy [29], meaning people who often feel joy at others’ bad luck tend to be less empathetic towards suffering. Low empathy could make one more comfortable with visiting a place of recent suffering without feeling as much distress or ethical conflict [14]. Additionally, someone high in schadenfreude might subconsciously view a disaster site visit as entertaining or exciting (in a way others might find inappropriate). In media psychology, for example, individuals prone to schadenfreude are more likely to seek out sensational news of others’ failures and even share such stories [28], suggesting a link to behavioral intentions (like forwarding an article) when misfortune triggers their interest. By analogy, visiting a disaster site could be seen as a form of experiential consumption of misfortune, fulfilling a similar drive. We thus propose that beyond the usual travel motives, a dispositional enjoyment of others’ misfortune can heighten one’s curiosity-driven intent to “go see” a place where tragedy befell others. In our study, we focus on the benign schadenfreude dimension – the kind of impersonal, low-level pleasure at seeing minor mishaps that many people might admit to (as opposed to malicious schadenfreude, which involves ill-will). This distinction is important because our context (a natural disaster) does not involve any perceived deservingness of victims; thus, any enjoyment would likely stem from curiosity at the spectacle rather than personal antagonism. We have clarified this focus in the manuscript. We thus propose two related effects of trait schadenfreude on the intention to visit disaster locations. First, a direct effect (H2): individuals higher in personal schadenfreude will report greater intention to visit a disaster site, all else being equal. This is our second hypothesis, formally stated:

*H2: Personal schadenfreude has a direct positive relationship with intention to visit disaster locations.*

This hypothesis aligns with dark tourism observations that some people are indeed motivated by the tragic aspect itself. If supported, it would indicate that beyond the usual travel motives, there is an incremental tendency for those who secretly enjoy misfortunes to want to “go see” a place where misfortune befell others.

### 2.5. The moderating effect of personal schadenfreude

Personal schadenfreude may amplify the influence of the standard motives on visit intention. In other words, someone high in schadenfreude who also has a curiosity motive, for example, might be especially likely to act on that curiosity by visiting a disaster site – more so than someone equally curious but low in schadenfreude. We expect a similar pattern for entertainment motive: an individual who generally seeks fun and excitement and who has a penchant for enjoying others’ mishaps might view a disaster site as an acceptable, even alluring venue for thrill, whereas a fun-seeking person with low schadenfreude might shy away out of respect or discomfort. In essence, schadenfreude could function as a lubricant that reduces the friction of moral restraint, allowing mainstream motives to translate into a disaster visit intention more freely. Those low in schadenfreude (especially if high in empathy) might self-censor or downplay their novelty or learning motives in this context due to sensitivity, whereas high-schadenfreude individuals feel less internal resistance to exploiting the site for their own interest. These moderation hypotheses were an important part of our theoretical framework. We anticipate interaction effects where schadenfreude strengthens the ‘motive to intention’ relationships. For example, the positive effect of novelty/curiosity on visit intention will be stronger among those high in schadenfreude (because their curiosity includes a fascination with the dramatic misfortune). Similar amplifying interactions are expected for entertainment and possibly education motives. Hence it is hypothesised that:

*H3: Personal schadenfreude positively moderates the relationship tourist motivation (novelty, fun, knowledge, and relationship) and intention to visit disaster locations.*

**Fig 1:**
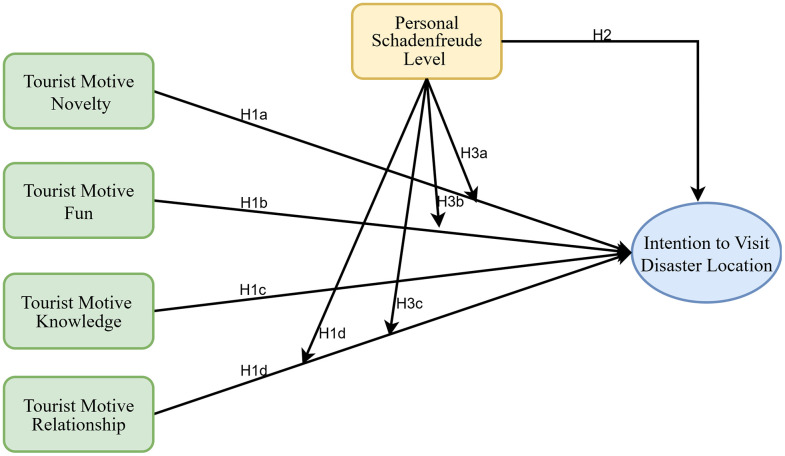
Proposed Theoretical Model.

We tested these moderations in our analysis, contributing a nuanced understanding of who (in terms of personality) is most prone to acting on various motives in a disaster tourism scenario. Fig 1 is the Proposed Theoretical Model.

## 3. Materials and methods

### 3.1. Research design and sample

This research employed a quantitative cross-sectional survey to test the proposed model in the context of visiting the 2024 Wayanad landslide sites. As the research asses post disaster visit intention and motivation to visit the land slide affected locations of Wayanad. The sampling unit (tourists/respondents) should be could meaningfully appraise the post-disaster visitation scenario and therefore purposive sampling was used to recruit the respondents. The target population was educated adults who were tourists to any of the three tourist spots in Kerala viz., Kovalam, Kumarakom, and Munnar; who were aware of the Wayanad landslides through media or personal connections. In order to ensure the minimum familiarity and understanding about the incident as screening question was asked to the tourists “Have you heard about the Wayanad landslide disaster in 2024?” and only those who are aware about the incident were only screened in to participate the survey. Participants were briefed with a short description of the disaster (to standardize knowledge) and then asked about their motives and intentions regarding a hypothetical visit to the landslide-affected area. The data was collected 4 months after the disaster. That is from 4^th^ December 2024–25^th^ January 2025. The timing was precisely chosen to capture intentions while the event remained relatively fresh in public memory, while immediate shock, restricted access, and emergency operations have abated. This period also coincided with recovery-stage communications (e.g., destination re-opening campaigns), indicating a context in which prospective travel deliberation becomes realistic.

An a priori power analysis was conducted using the A-priori Sample Size Calculator for Structural Equation Models (SEM) [30] to determine the minimum required sample size for the proposed model. Inputs reflected a medium anticipated effect size (f² = .30; [31]), α = .05, desired power = .90, 6 latent variables, and 22 observed indicators. The calculator returned (a) a minimum N = 200 to detect the specified effect with 90% power at α = .05, and (b) a minimum N = 123 to accommodate the model’s structural complexity [32]; hence, the recommended minimum N was 200. However, considering the response rate and time gap in obtaining the data we have distributed 600 questionnaires, out of which 453 were completely filled and returned. After running the initial screening and cleaning only 438 responses found useful and our final sample (N = 438) exceeded these thresholds, supporting adequate power for hypothesis testing in a variance-based SEM framework and aligning with lower-bound guidance for SEM sample sizes [33]. The data set supporting the research finding were provided alongside with the manuscript and supplementary material.

Ethical approval for the study was obtained from the university’s Institutional Review Board. All participants were 18 years of age or older. Participation was voluntary and anonymous. Potential participants were informed about the study’s purpose, associated risks, and benefits. The respondents were assured that they could decline or discontinue at any time without penalty. For recruitment of the respondents, tourists were first approached in person at the field sites viz., Kovalam, Kumarakom, and Munnar. Those who agreed to participate were given a standardized explanation of the study and completed a written informed consent form on site physically. Subsequently, a secure link to the online questionnaire (using Qualtrics) was sent to consenting participants via email or WhatsApp later own at their convenience preferred at the time of filling up the participant information sheet. Informed consent was therefore obtained prior to any data collection, and data were collected only from individuals who had provided consent.

### 3.2. Measures

All constructs were measured with validated multi-item scales, adapted slightly to fit the Wayanad context. Unless otherwise noted, items used a 5-point Likert agreement scale (1 = strongly disagree, 5 = strongly agree). Tourist Motives were operationalised as four separate latent variables viz., Novelty/Curiosity, Entertainment/Fun, Education/Learning, and Relationship motives – corresponding to the theoretical categories in H1. For each motive, 3–5 items were adapted from established tourism motivation scales in the literature (e.g., the motivation battery used by Correia, Kozak & Ferradeira [14], Pearce & Lee [34] and the intrinsic motivation measures in Yoo, Yoon & Park [17]). Example items include: “I am interested in visiting places that are new and different” (Novelty motive); “I travel to have fun and enjoyment” (Entertainment motive); “I like to learn about events or places that I visit” (Education motive); and “Traveling is a way for me to bond with my friends/family” (Relationship motive). Respondents were asked to rate how much each reason applied to “visiting the Wayanad landslide site in the future.” Tying the items to the specific context ensured that the motives were evaluated in light of that destination (rather than travel in general). This approach follows the logic of prior studies which tailor general motivation items to a particular destination or scenario [19]. Table 1, shows the measurement model statistics. The motive scales demonstrated good reliability in this study (See Table 1, Cronbach’s α ranging.985–.990 for the four scales). A confirmatory factor analysis supported their distinctiveness, consistent with theories that novelty, social, knowledge, etc., are related but distinguishable motivational factors [15].

**Table 1:** Measurement Model Statistics.

Construct	Indicator	TMN	SDF	TMF	TMK	TMR	INV	alpha	rhoC	AVE	rhoA
**Tourist Motive Novelty (TMN)**	TMN1	0.993	–	–	–	–	–	0.990	0.993	0.981	0.990
	TMN2	0.987	–	–	–	–	–				
	TMN3	0.990	–	–	–	–	–				
**Tourist Motive Fun (TNF)**	TMF1	–	–	0.984	–	–	–	0.986	0.991	0.973	0.994
	TMF2	–	–	0.991	–	–	–				
	TMF3	–	–	0.985	–	–	–				
**Tourist Motive Knowledge (TMK)**	TMK1	–	–	–	0.986	–	–	0.985	0.990	0.971	0.986
	TMK2	–	–	–	0.985	–	–				
	TMK3	–	–	–	0.986	–	–				
**Tourist Motive Relationship (TMR)**	TMR1	–	0.991	–	–	–	–	0.988	0.992	0.976	0.988
	TMR2	–	0.985	–	–	–	–				
	TMR3	–	0.988	–	–	–	–				
**Personal Schadenfreude Level (SDF)**	SDF1	–	–	–	–	0.865	–	0.920	0.938	0.716	0.932
	SDF2	–	–	–	–	0.881	–				
	SDF3	–	–	–	–	0.725	–				
	SDF4	–	–	–	–	0.834	–				
	SDF5	–	–	–	–	0.890	–				
	SDF6	–	–	–	–	0.874	–				
**Intention to Visit Disaster Location (INV)**	INV1	–	–	–	–	–	0.908	0.918	0.942	0.803	0.920
	INV2	–	–	–	–	–	0.899				
	INV3	–	–	–	–	–	0.903				
	INV4	–	–	–	–	–	0.875				

The present study uses the benign subscale because it taps every day, impersonal enjoyment of minor misfortunes and therefore aligns with curiosity-oriented engagement at a post-disaster site; the malicious dimension, by contrast, centres on ill-will toward disliked others. Using the benign subscale reduces social-desirability concerns and participant discomfort while keeping the instrument brief for a field setting. Personal Schadenfreude was measured using the benign schadenfreude subscale of the Trait Schadenfreude Scale developed by Crysel & Webster [28]. The full scale has 12 items (6 per dimension benign/malicious); we used the 6 benign items, which capture a general tendency to take mild pleasure in others’ misfortunes without malicious intent. These items were presented as statements such as “I sometimes feel a bit of enjoyment when I hear about minor bad luck happening to others” and “When I see a news story about a mishap (with no serious harm done), I find it interesting or amusing.” Respondents indicated agreement on the 5-point scale. The scale’s authors report that benign schadenfreude reflects commonplace reactions like laughing at slapstick accidents [28], as opposed to ill-wishing harm. In our data (See, Table 1), the schadenfreude items showed good internal consistency (α = .920). It is worth noting that trait schadenfreude has been shown to be inversely related to empathy [29]; while we did not measure empathy directly, this characteristic of the construct underlines why high schadenfreude individuals might be more inclined to engage with a disaster site (less empathetic inhibition). We scored the schadenfreude scale so that higher values represent a greater dispositional enjoyment of others’ misfortune.

Intention to Visit Disaster Site was the primary dependent variable. We measured visit intention with behavioural intention measure developed by Meng & Choi [35]. The items were: (1) “I intend to travel to Wayanad landslide site in the near future,” (2) “I am planning to travel to Wayanad landslide site in the near future,” (3) “I will make an effort to Wayanad landslide site in the near future,” and (4) “I will certainly invest time and money to travel Wayanad landslide site in the near future.” These items were averaged to form an intention index (See Table 1, α = .918). Although visiting a disaster site is an unusual behaviour, framing the intention in terms of interest and likelihood is consistent with how visit intentions to new or unique destinations are assessed in tourism [17]. The items were worded carefully to avoid sounding morbid; they focused on the act of visiting and seeing the affected area, not on enjoying others’ suffering. Nonetheless, a high score on this scale indicates a person is positively inclined to engage in “disaster tourism” in this case.

Additionally, we collected relevant control variables such as age, gender, educational qualification, occupation, Nationality and Zone of residence to expose the characteristics of the respondents and to be controlled if needed. We also asked a question about the moral judgment of disaster tourism (“I believe it is okay for tourists to visit the Wayanad landslide site as a tourist attraction”) to gauge whether ethical stance might confound intention; interestingly, responses on this moral approval item correlate with schadenfreude levels, providing some face validity that those with higher schadenfreude are less opposed to such tourism ethically.

### 3.3. Analytical procedure

The data is analysed using Partial Least Squares Structural Equation Modelling (PLS-SEM), with SEMINR package in R. PLS-SEM was chosen for several reasons. First, our research is predictive and exploratory in nature, we aim to explain variance in visit intention and test interaction effects, an area where PLS-SEM excels due to its prediction-oriented approach. Second, the model includes multiple latent constructs and an interaction term (motivation × schadenfreude), which can be handled flexibly in PLS. Third, PLS-SEM places minimal demands on data in terms of distribution and sample size [36]. It is a variance-based SEM technique that uses ordinary least squares estimation, yielding robust results even with complex models and without strict normality – appropriate for our sample of 438, which while decent in size, benefits from PLS’s efficiency in model estimation [37]. In technical terms, PLS SEM builds the measurement and structural model simultaneously and is well-suited for theory development stages. According to Hair et al. [38] PLS-SEM can attain high levels of statistical power with relatively lower sample sizes than covariance-based SEM, and it is ideal when the research objective is explaining variance (R²) in target constructs (here, intention) rather than confirming a well-established theory. These conditions align with our study’s goals.

## 4. Results

The modelling proceeded in two stages: a measurement model assessment and a structural model assessment. As the research model is built on PLS-SEM, normality is not a matter for concern [39]. The variance inflation factor of all the items of all the construct was under 5, indicating noncollinearity [39]. The study has employed same method of data collection for independent as well as dependent variable, there exists the possibility of common method bias [40]. Therefore, we also checked for common method bias using Harman’s single-factor test (no single factor explained the majority of variance) and by incorporating a measured marker variable; results suggested that single variable can only explain 38% of variance, indicating that common method variance was not a serious concern [41].

### 4.1. Respondents Characteristics

Out of 438 respondents in the study, nearly half were between 30 and 45 years old (n = 209, 47.7%), 42.7% were under age 30 (n = 187), and 9.6% were over age 45 (n = 42). Gender distribution was fairly balanced: 42.0% identified as female (n = 184), 37.0% as male (n = 162), while 0.9% identified as non-binary or third gender (n = 4); 20.1% (n = 88) preferred not to disclose their gender. Marital status was dominated by never-married respondents (65.8%, n = 288); 21.7% were married (n = 95), 7.8% divorced (n = 34), and 4.8% widowed (n = 21). Educational attainment varied: 39.95% held an undergraduate degree or equivalent (n = 175), 23.74% had completed a pre-degree or +2 level education (n = 104), 20.55% had up to matriculation (n = 90), and 15.75% held a postgraduate degree or higher (n = 69). Regarding occupation, the largest group was not working (43.6%, n = 191), followed by those in private employment (29.2%, n = 128), self-employed individuals (17.8%, n = 78), and government employees (9.4%, n = 41). Finally, monthly income was distributed with 45.2% earning between ₹25,000 and ₹50,000 (n = 198), 28.8% earning up to ₹25,000 (n = 126), and 26.0% earning between ₹50,000 and ₹75,000 (n = 114).

### 4.2. Measurement Model Evaluation

In the measurement model, we verified indicator loadings, internal consistency reliability (Cronbach’s α and composite reliability), convergent validity (Average Variance Extracted, AVE) and discriminant validity (Fornell-Larcker criterion, cross-loading checks, and Heterotrait-Monotrait HTMT) for all latent constructs. As shown in Table 1, all the motivational constructs, schadenfreude construct and the intention to visit construct has Cronbach’s α value as well as rhoC value over and above 0.70 indicating the internal consistency reliability [38]. Similarly, all motivation constructs and the schadenfreude construct exhibited loadings above 0.70 on their intended factors and has no observations of cross loading confirming the discriminant validity of the constructs in between [39] and AVE above the 0.50 threshold, indicating that the measures were sound. For example, AVE for Novelty motive was 0.98, for Fun motive 0.97, etc., The discriminant validity certain (Fornell-Larcker) shown in Table 2, confirms that the latent inter-correlations were below the square roots of AVEs, supporting discriminant validity. The trait schadenfreude items loaded strongly on a single factor (the square roots of AVE = 0.85), separate from any motive factors, as expected – confirming that schadenfreude is empirically distinct from, say, simply having a curiosity motive [42]. Furthermore, the Table 3 shows that as a collaborative to FL and cross loading criterion validity, the HTMT values of all the motivational, schadenfreude and intention to visit construct were well under the HTMT threshold of 0.80 [43] cross validating the discriminant validity.

**Table 2:** Fornell-Larcker Criterion Statistics.

Construct	TMN	SDF	TMF	TMK	TMR	INV
TMN	0.990	–	–	–	–	–
SDF	0.421	0.846	–	–	–	–
TMF	−0.010	−0.098	0.987	–	–	–
TMK	0.091	0.347	0.188	0.985	–	–
TMR	0.321	0.400	0.199	0.458	0.988	–
INV	0.488	0.659	−0.090	0.425	0.471	0.896

**Table 3:** HTMT Criterion Statistics.

Construct	TMN	SDF	TMF	TMK	TMR
TMN	–	–	–	–	–
SDF	0.437	–	–	–	–
TMF	0.014	0.138	–	–	–
TMK	0.092	0.367	0.190	–	–
TMR	0.324	0.421	0.201	0.464	–
INV	0.510	0.707	0.094	0.447	0.495

### 4.3. Structural Model Evaluation

**Fig 2:**
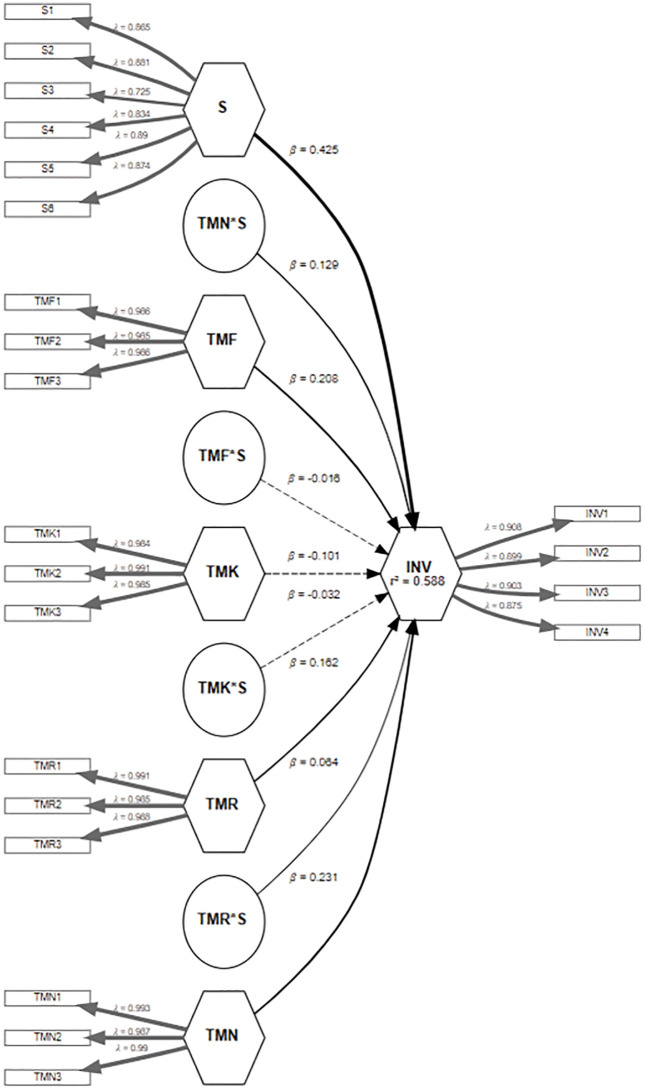
Structural Model Explaining the Relationship Between the Constructs.

For the structural model, we included paths from each motive construct to Visit Intention, the direct path from Schadenfreude to Visit Intention (testing H2), and interaction terms for Schadenfreude × (each motive) to Visit Intention (to examine moderation effects underpinning H1’s moderation aspect). The interaction terms were created in PLS using standardized product indicators. We used bootstrapping (5,000 resamples) to assess path coefficients’ significance. The structural model statistics were shown in Table 4. The model fit indices in PLS (such as SRMR) were within acceptable ranges (SRMR = 0.061), though exact model fit is secondary in PLS; more importantly, as per Fig 2 the R² for Visit Intention was 0.588, indicating the model explains about 58.80% of the variance in intention, which is substantial for this type of behaviour. We also examined f² effect sizes for each predictor and interaction to gauge their relative contribution.

**Table 4:** Structural Model Statistics.

Hypothesis	Relationship Path	B	β	SE β	T Stat.	5% CI	95% CI	Status of Hypothesis
H1a	TMN → INV	0.231	0.229	0.040	5.743	0.164	0.293	Supported
H1b	TMF → INV	−0.101	−0.100	0.041	−2.486	−0.165	−0.033	Supported
H1c	TMK → INV	0.208	0.204	0.047	4.483	0.126	0.278	Supported
H1d	TMR → INV	0.162	0.166	0.041	3.930	0.101	0.234	Supported
H2a	SDF → INV	0.425	0.427	0.047	9.045	0.348	0.504	Supported
H3a	TMN × SDF → INV	0.129	0.129	0.042	3.107	0.059	0.196	Supported
H3b	TMF × SDF → INV	−0.032	−0.029	0.029	−1.108	−0.075	0.019	Not Supported
H3c	TMK × SDF → INV	−0.016	−0.019	0.045	−0.360	−0.094	0.058	Not Supported
H3d	TMR × SDF → INV	0.064	0.072	0.063	1.014	−0.032	0.170	Not Supported

The direct effects of the four tourist motives and personal schadenfreude (SDF) on intention to visit (INV) are summarized in Table 4. In the structural model, novelty-seeking emerged as a significant positive predictor of intention to visit a disaster location, B = 0.231, β = .229, SE = 0.040, t (437) = 5.74, 95% CI [0.164, 0.293], indicating that greater desire for novel experiences is associated with stronger visit intentions. Personal schadenfreude showed a robust positive association with visit intention, B = 0.425, β = .427, SE = 0.047, t (437) = 9.05, 95% CI [0.348, 0.504], suggesting that individuals who derive more benign pleasure from others’ misfortunes are more inclined to visit a disaster site. In contrast, the fun motive was a small but significant negative predictor of visit intention, B = −0.101, β = −.100, SE = 0.041, t (437) = −2.49, 95% CI [−0.165, −0.033], indicating that those primarily motivated by entertainment are slightly less likely to plan such a visit. The knowledge motive positively predicted intention, B = 0.208, β = .204, SE = 0.047, t(437) = 4.48, 95% CI [0.126, 0.278], and the relationship motive also had a modest positive effect, B = 0.162, β = .166, SE = 0.041, t (437) = 3.93, 95% CI [0.101, 0.234], indicating that those seeking learning or social bonding are more likely to express an intention to visit a disaster-affected area.

### 4.4. Moderating Effect of Schadenfreude in the Relationship Between Tourist Motive and Intention to visit Disaster Location

**Fig 3:**
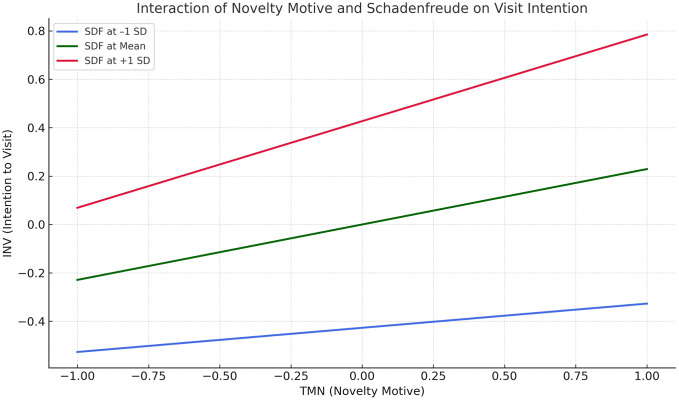
Moderation of Schadenfreude in the Relationship Between Tourist Motive (novelty) and Intention to Visit Disaster Location.

A moderation analysis examined whether personal schadenfreude (SDF) altered the strength of each tourist motive’s effect on intention to visit (INV). Only the interaction between novelty motive and schadenfreude was significant, B = 0.129, β = .129, SE = 0.042, t (437) = 3.11, 95% CI [0.059, 0.196], indicating that the positive relationship between novelty‐seeking and visit intention was stronger at higher levels of schadenfreude. The interactions for fun motive (B = –0.032, β = –.029, SE = 0.029, t = –1.11, 95% CI [–0.075, 0.019]), knowledge motive (B = –0.016, β = –.019, SE = 0.045, t = –0.36, 95% CI [–0.094, 0.058]), and relationship motive (B = 0.064, β = .072, SE = 0.063, t = 1.01, 95% CI [–0.032, 0.170]) were not significant, suggesting schadenfreude did not meaningfully change how these motives related to the intention to visit a disaster location. Figure 3 displays the simple-slope analysis for the interaction of novelty motive (TMN) and personal schadenfreude (SDF) on intention to visit a disaster location (INV). Three regression lines represent TMN at one standard deviation below the mean of SDF (blue), at the mean (green), and one standard deviation above the mean (red). Across all SDF levels, higher TMN scores are associated with higher INV scores; however, the slope steepens as SDF increases. The line is flattest when SDF is low, steeper at the mean, and steepest at high SDF, visually confirming the significant TMN × SDF interaction reported in Table 4 (β = .129, t = 3.11, 95% CI [.059,.196]). The upward displacement of the lines further indicates that, at any given level of novelty seeking, individuals with greater schadenfreude report higher intentions to visit the disaster site.

## 5. Discussion

This research intended to examine how mainstream tourist motivations viz., novelty, education, entertainment, and relationship alongside with the personality trait of benign schadenfreude influence individuals’ intention to visit a recent disaster site. The measurement models validate the model dimension as well as the factor structure. Whereas, the structural model provide strong support for the hypothesized relationships, offering valuable theoretical and practical insights into the dynamics of post-disaster tourism.

### 5.1. The Role of Mainstream Tourist Motives

As hypothesised (See, hypothesis 1), three of the four conventional travel motives viz., novelty seeking, knowledge seeking, and relationship building have significant positive association with associated with intention to visit the disaster site. Whereas the relationship between the entertainment and intention to visit was significant but negative. This indicates that, travellers who travel for the purpose of fun-seeking experience were less supposed to visit a disaster location. Visiting a location of landside is not a relaxing destination. Therefore, the tourists who travel for with the purpose of amusement experience are supposed to stay away from the disaster locations. However, the research underscores that while mainstream travel motives viz., novelty seeking, knowledge seeking, and relationship building can be carried over into the context of disaster tourism also. But the entertainment motive, grounded on fun and entertainment experience can not only be translated into a disaster location visit but also deter fun seeking tourists from the disaster sites, considering the solemn nature of disaster sites.

While considering the conventional tourist motives individually, the motive of novelty seeking has the strongest association with intention to visit disaster location (β = .229, t = 5.74), suggesting that the uniqueness of the disaster landscape may trigger curiosity and desire among novelty-driven tourists to visit the disaster location to experience the uniqueness of the disaster location, though it is an outcome of a disaster. This is because the novelty, irrespective of its nature is identified as a core “push” factor [11,12]. The motive of knowledge seeking also has a significant role (β = .204, t = 4.48), indicating that tourists with academic or informational motives perceive value in visiting the disaster site for the purpose of observing and analysing the physical and social consequences of the disaster. This is what the Crompton and Biran et al. [15,19] identified educational and commemorative motives often drive visits to heritage or dark tourism sites. This is because the physical site serves as a ‘reality check’ that validates media reports, allowing visitors to construct a coherent narrative of the event through actual visit. That is, the intention to visit is driven by a need to process the tragedy intellectually rather than emotionally. Further, the relationship motive, that is, where the travellers identify the tourist destinations as an opportunity to share and revamp social bonds and connections [15,17], exhibited a moderately positive and significant association with intention to visit disaster location (β = .166, t = 3.93). The group visit to disaster location may serve a relational function, enabling collective reflection or storytelling [15,17]. Moreover, the disaster site acts as a unique backdrop that fosters ‘communitas’ a sense of shared solidarity in the face of adversity, which motivates peoples and groups to visit the location as a way of reinforcing their social connections through a distinct, shared memory. In contrast, the fun/entertainment motive was negatively associated with intention to visit a disaster location (β = –.100, t = –2.49). This outcome is consistent with the conception that disaster sites, characterized by solemnity and loss, are misaligned only with the hedonistic expectations of fun-seeking tourists. Such environments likely lack the light hearted appeal typical of leisure travel, thus deterring those motivated by play or relaxation.

### 5.2. Direct and moderated effects of personal schadenfreude

The analysis validates the Hypothesis 2, that there is significant positive association between personal schadenfreude and disaster-site visit intention (β = .427, t = 9.05), out of various predictors for disaster-site visit intention the personal schadenfreude was identified as the strongest. The positive association between personal schadenfreude and disaster-site visit intention, affirms that the individuals scoring high on benign schadenfreude were significantly more inclined to express interest in visiting the landslide-affected area. This research finding was an empirical validation of the theoretical framework that schadenfreude may operate as an independent “dark” motive for thanatourism [4,26,28]. Further, introducing benign schadenfreude as a moderator between the tourist motive and intention to visit disaster location add lucidity to the operative framework of the personal schadenfreude. As per the moderation analysis, the interaction between novelty seeking and schadenfreude was statistically significant for intention to visit disaster location (β = .129, t = 3.11). This validates the Hypothesis 3a that individuals with high levels of novelty motive and schadenfreude exhibited especially strong intentions to visit the disaster location. This significantly positive interaction suggests that, schadenfreude acts as a psychological catalyst, in the relationship between novelty motive and intention to visit disaster location. The personal schadenfreude (finding joy in others misfortune) diminishes the moral inhibitions, resulting the transformation of curiosity into concrete behavioural intent. Whereas, tourists with lower schadenfreude, in despite of their high novelty orientation, may experience greater empathic resistance that tempers visit intentions. It may because of this, even at high levels of novelty motive, tourists with low level of schadenfreude exhibits little interest to visit the disaster site. However, while considering the other conventional tourist motive dimensions viz., entertainment (β = −0.029, t = −1.108), knowledge (β = −0.019, t = −0.360), relationship (β = 0.072, t = 1.014)., schadenfreude has no moderating effect on intention to visit disaster location. This finding is an indication that the personal schadenfreude can only amplify tourist motive (novelty) but it does not have the potential to exert influence of other motivational domains. Fun-seeking travellers appear disinterested in disaster sites regardless of schadenfreude, likely due to contextual incongruity. Knowledge- and relationship-oriented travellers pursue their goals through respectful learning and bonding, rendering schadenfreude irrelevant to their decision-making process.

### 5.3. Theoretical implications

The research makes several theoretical contributions. First, this research empirically validates the applicability of mainstream travel motives viz., entertainment, relationship, novelty, knowledge., in the context of dark tourism. Further, the research validates the theoretical preposition that rather than being driven solely by morbid curiosity or voyeurism, visitors to disaster sites may be motivated by curiosity, education, or social connection, echoing motives commonly observed in heritage or cultural tourism [15,19]. This challenges the prevailing notion with the help of real-world data that the disaster tourism is always predominantly “dark” in its psychological underpinnings. Second, the study extends the understanding of individual differences by demonstrating that trait-level schadenfreude can both independently and interactively shape tourism intentions from a broader perspective. Interestingly, this finding doesn’t alienate itself rather it aligns with existing literature that the individuals high in benign schadenfreude exhibit lower empathy [26], making them more receptive to contexts others might find emotionally challenging. Similarly, the findings effectively exhibit the interaction between schadenfreude and novelty and also corroborates with psychological theories suggesting that moral emotions can modulate exploratory behaviour in individuals which in turn result in the reduced empathic inhibitions and may embolden actions typically constrained by social norms. Third, this research presents a more differentiated conceptual motivation model for disaster the tourism by introducing the personality trait-based dispositions (schadenfreude) alongside with situational motives to explain the intentions behind disaster tourism. The study effectively explained how affective personality frameworks with behavioural intention theory in tourism. The proposed model of disaster tourism suggests that dark tourism behaviour is not just monolithically driven by pathology or spectacle, but by the interplay of ordinary motivations and affective predispositions. Our findings also align with broader evidence that enduring dispositions and value orientations map onto concrete behavioural outcomes across domains. For example, cultural–value dispositions have been shown to predict low-carbon behaviours, and cognitive-style dispositions to predict cyberloafing in organizational settings. Such parallels reinforce the theoretical move of integrating trait-level affective/cognitive predispositions with situational motives when explaining intention formation.

Our findings can be embedded within broader destination image and place identity frameworks. First, if disaster-site image shapes behavioural outcomes via authenticity and emotional experience, as shown by Xu et al. [44], then the learning and novelty pathways we observe may be partially mediated by those affective/appraisal mechanisms. Second, consistent with Dai et al. [45], place identity could function as a mediator linking knowledge-seeking and relationship motives to longer-term outcomes (e.g., revisit or advocacy), especially when narratives of resilience foster identification with the destination. Future work should explicitly test these mediated pathways alongside our schadenfreude moderation [44,45]. Theoretically, this study extends the Push-Pull Motivation Framework into the domain of dark tourism and linking it with trait activation theory to explain the visit intensions. While ‘push’ factors (Novelty, Learning) typically drive intention, this research demonstrates that these mechanisms are not isolated. By validating Trait Activation Theory in this context, we show that how the personality trait of schadenfreude acts as a ‘release mechanism’ that allows latent curiosity to manifest as visit intention, effectively bridging the gap between general travel motivation and dark tourism behaviour.

### 5.4. Practical implications

From a managerial perspective, the findings have important implications for the design and governance of tourism experiences in post-disaster contexts. Most notably, site managers should recognize the heterogeneity in visitor motives. Many individuals are drawn to such sites for reflective, educational, or relational reasons desires that can be constructively supported through interpretive signage, guided tours, or memorial elements that emphasize learning and remembrance [5,16].At the same time, the evidence that some visitors are motivated, at least in part, by a covert enjoyment of misfortune underscores the need for ethical management. Without appropriate guidelines, these motives may manifest in insensitive behaviours such as taking selfies or treating the site as a spectacle which can distress affected communities and damage the site’s integrity. Ethical visitor codes, educational framing, and staff training can help shape respectful engagement and mitigate voyeuristic tendencies [20]. Moreover, understanding the motivational profile of prospective visitors enables tourism planners to better anticipate demand and align marketing strategies accordingly. For example, messaging that highlights opportunities for learning and commemoration may resonate with novelty- and knowledge-oriented travellers, while downplaying sensationalism that might attract inappropriate interest. Importantly, planners should avoid framing disaster tourism as “thrill-seeking,” even if some visitors interpret it that way. The destination marketing organizations and local authorities might consider using post-disaster tourism as a tool for regional recovery so long as it is managed with cultural sensitivity and community consultation. By channelling tourism toward remembrance and education, rather than spectacle, it is possible to support both healing and economic regeneration.

Managers of disaster sites should avoid assuming that all visitors are morbid thrill‑seekers. Evidence shows that many tourists are motivated by curiosity, learning, remembrance, and meaning‑making, which can be addressed through educational and commemorative programming at the site [46,47]. At the same time, the presence of voyeuristic or schadenfreude‑driven interest highlights the need for ethical guidelines and sensitive management [47]. Tourism planners and local authorities should anticipate that a subset of visitors may approach with limited empathy and implement clear codes of conduct, interpretive signage, and guided experiences to set appropriate norms [23,24].Balancing pedagogical value with respect for suffering is essential if such sites are to be opened to tourism in socially responsible and sustainable ways [48]

## 6. Conclusion

This study contributes to the understanding of post-disaster tourism by empirically demonstrating that mainstream travel motives especially novelty, learning, and social bonding substantially explain why people intend to visit a recent disaster site, while a purely fun-seeking orientation deters such interest. Importantly, we establish that a benign form of schadenfreude not only directly increases the likelihood of disaster-site visitation but also amplifies the effect of novelty-seeking on visit intentions. By integrating a psychological trait into a tourism motivation model, our work bridges conventional tourist behaviour theory with the dark tourism context, offering a more nuanced view of what drives visitation to tragedy sites. From a practical perspective, the findings suggest that managers of disaster sites should not assume all visitors are morbid thrill-seekers. Many tourists are driven by curiosity, a desire to learn, or a wish to pay respect and gain understanding – motives that can be constructively addressed through educational and commemorative initiatives at the site. At the same time, the evidence of schadenfreude-driven interest highlights the need for ethical guidelines and sensitive management. Tourism planners and local authorities should prepare for the possibility that a subset of visitors may approach the site with less empathy and more voyeuristic intent. Clear codes of conduct, interpretive signage, and guided programs can help set the appropriate tone, ensuring that visitor engagement remains respectful. Balancing educational value with respect for suffering is essential if such sites are to be opened to tourism in a sustainable and socially responsible manner [20].

Finally, we acknowledge several limitations of this research. The study relied on a purposive sample of Indian respondents familiar with one specific disaster, which may limit generalizability to other cultural contexts or types of catastrophes. The cross-sectional survey design also precludes any firm conclusions about causality. Additionally, our focus was restricted to benign schadenfreude, more malicious forms of schadenfreude or other dark personality traits were not measured, potentially underestimating the influence of darker impulses on disaster tourism interest. Future research should therefore replicate and extend this work in other settings, including different cultural contexts and both natural and human-made disaster scenarios – and employ longitudinal or experimental designs to test causal relationships. It would be valuable to examine additional individual differences (e.g., empathy, moral identity, sensation-seeking) that might interact with tourist motives. In particular, assessing the role of malicious schadenfreude could reveal whether a more malevolent pleasure in others’ suffering further drives interest in dark tourism [4]. Addressing these questions in future studies will deepen theoretical insight into dark tourist behaviours and inform more effective and ethical management strategies for destinations recovering from catastrophic events.

## Supporting information

S1 Table Essential Data. (XLSX)
